# Tomographic 3D Ultrasound for Thyroid Volumetry—A Comparison Between Multiplanar 2D and 3D Imaging Methods

**DOI:** 10.3390/diagnostics16152329

**Published:** 2026-07-25

**Authors:** Robert Roth, Zoe Reinke, Martin Siedlecki, Jan Luitjens, Martin Hügle, Johanna S. Enke, Christian Liska, Laura-Marie Feitelson, Constantin Lapa, Thomas Kroencke, Robert Bauer, Thomas Wendler

**Affiliations:** 1 PIUR Imaging GmbH, 1070 Vienna, Austria; 2 Diagnostic and Interventional Radiology and Neuroradiology, Faculty of Medicine, University of Augsburg, 86156 Augsburg, Germany; 3 Digital Medicine, Faculty of Medicine, University of Augsburg, 86156 Augsburg, Germany; 4 Nuclear Medicine, Faculty of Medicine, University of Augsburg, 86156 Augsburg, Germany; 5 Bavarian Cancer Research Center (BZKF), 86156 Augsburg, Germany; 6 Center for Advanced Analytics and Predictive Sciences (CAAPS), University of Augsburg, 86159 Augsburg, Germany; 7 Computer-Aided Medical Procedures and Augmented Reality, School of Computation, Information and Technology, Technical University of Munich, 85748 Garching, Germany

**Keywords:** thyroid volumetry, tracked 3D ultrasound, interobserver variability, reproducibility, multi-modality phantom, ellipsoid method, tissue-mimicking material

## Abstract

**Background:** Accurate thyroid volume measurement is essential for diagnosing and monitoring thyroid disorders and for determining radioiodine therapy dosage. The ellipsoid method applied to two-dimensional B-mode ultrasound (Bmode-2D-US) is the most prevalent clinical approach, yet it is known to suffer from high observer dependence and limited accuracy. Tracked three-dimensional ultrasound (3D-US) is a promising radiation-free alternative, but direct comparisons across modalities under controlled conditions remain scarce. **Methods:** A custom-built anthropomorphic multi-modality phantom was constructed containing six anatomically realistic thyroid lobe samples molded from segmented MRI data, with ground truth volumes verified by the suspension method and 3D surface scanning. Volume measurements were performed by six observers of varying experience using Bmode-2D-US, electromagnetically tracked 3D-US (EM-3D-US), inertial-measurement-unit-tracked 3D-US (IMU-3D-US), and optically tracked 3D-US (Opt-3D-US), as well as computed tomography (CT) and magnetic resonance imaging (MRI). Inter- and intraobserver variability were assessed using modified Bland–Altman analysis. **Results:** All three 3D-US methods reduced inter- and intraobserver variability compared to Bmode-2D-US, achieving variability comparable to CT and lower than MRI. Mean accuracy was similar across 3D-US, CT, and MRI. Bmode-2D-US showed strong observer dependence, with operator experience having the most pronounced effect on this method. Among tracked methods, EM-3D-US and Opt-3D-US were least sensitive to operator movement quality, while IMU-3D-US showed somewhat higher sensitivity. **Conclusions:** Tracked 3D-US is a promising radiation-free alternative to conventional Bmode-2D-US for thyroid volumetry, offering improved reproducibility and accuracy across operators of varying experience in this phantom-based evaluation.

## 1. Introduction

The volume of thyroid lobes and thyroid nodules are both common clinical indicators for thyroid pathologies, relied upon in diagnosis and treatment. Precise volumetry can allow the identification and tracking of volume changes over time, and can be used at several stages in the interventional therapy of thyroid pathologies such as thermal ablation or high-intensity focused ultrasound. Beyond precision for the evaluation of volume changes, achieving absolute accuracy in the volume measurements can be useful to determine the correct dosage in radioactive iodine therapy, since the dosage is directly dependent on the thyroid volume [1]. For this reason, the ellipsoid method on ultrasound has become the most prevalent approach. Therein, two-dimensional measurements from B-mode ultrasound (Bmode-2D-US) in two perpendicular planes (axial and sagittal) are used to extrapolate a volume approximation for thyroid lobes or thyroid nodules. It is reported to have both high variation and high dependence on the measuring clinician [2,3].

When determining thyroid nodule growth with the ellipsoid method, only a measured increase of 50% is recommended to be read as significant in clinical decision-making [4]. Critically, it has been noted that any measured increase smaller than this threshold cannot reliably be interpreted as actual growth, since differences between follow-ups may result from measurement variability alone [5]. Higher precision and accuracy could improve decision-making, but remain limited by the low quality of standard volume estimation, so the accuracy and reproducibility of these techniques must be improved. Several technologies are promising to fulfil this requirement. While computed tomography (CT) is a highly established technology in medical imaging, radiation exposure and costs limit its suitability for routine use. Magnetic resonance imaging (MRI) does not require ionizing radiation, but is even more costly and has additional exclusion criteria. Three-dimensional tracked ultrasound imaging (3D-US) shows potential to be a better fit for the volumetry in thyroid imaging.

The goal of the present study is to compare the quality of thyroid lobe volume measurements across several medical imaging technologies.

### 1.1. State of the Art: Thyroid Volume Measurement

A number of research groups have previously compared the quality of volume measurement methods, particularly in comparison to ultrasound.

Lyshchik et al. [3] determined accuracy and intraobserver variability of thyroid volume measurements for both the ellipsoid method in Bmode-2D-US and electromagnetically tracked 3D ultrasound (EM-3D-US). Pre-surgery measurements were conducted three times for each of 47 children with planned thyroidectomy to determine thyroid volume. After surgery, a reference volume was measured by weighing the resected thyroids, assuming a density of 1 g/cm^3^. EM-3D-US was found to have superior accuracy and more consistent measurements than Bmode-2D-US.

Freesmeyer et al. [6] compared Bmode-2D-US, EM-3D-US, CT and magnetic resonance imaging (MRI). A thyroid phantom was constructed with the goal of achieving a high-accuracy reference volume. The thyroid was imitated with a balloon filled with different volumes of water and contrast medium, submerged in a water bath. The phantom was intentionally deformed into three shape variants using cable ties on the balloon. EM-3D-US, CT and MRI achieved similarly good results, while Bmode-2D-US was less accurate.

Krönke et al. [7] measured the volume of thyroid lobes using 2D and EM-3D-US combined with a convolutional neural network. The interobserver variability between medical doctors using EM-3D-US was lower than when using Bmode-2D-US. The accuracy of both methods was also evaluated by comparing them to MRI manual segmentation as the reference. Results showed that EM-3D-US is more consistent with MRI.

### 1.2. Limitations of the State of the Art

A common limitation found in the state of the art is the limited number of different volumetry technologies that are compared within the same study. In phantom studies, this can be partially attributed to the added complexity in building multi-modality phantoms. In patient studies, ethical issues prevent performing a high number of medically unnecessary imaging procedures on the same patient. Further reasons include setup complexity, simultaneous availability of technologies, and prolonged completion time for observers.

A direct reference value for the true thyroid volume is not achievable for patient studies unless the thyroid is removed for direct volume measurement. However, such an approach poses some difficulties in getting highly accurate results, as complete thyroid resection might not always be achievable and the borders for measurement in vivo might not exactly match the borders of resection of individual lobes.

To get a high-quality volume reference, phantom building is useful as it also permits direct measurement of the true volume. Nevertheless, care must be taken to realistically represent anatomical shapes. The use of fluid-filled balloons in phantom building, as applied by Freesmeyer et al. [6], allows accurately filling specified reference volumes, but also gives a very smooth surface resembling ellipsoids. This shape likely contributes to biasing results where the ellipsoid method might perform better than should be expected for realistic anatomical thyroid shapes. Despite this possible shape bias, it is notable that the study by Freesmeyer et al. [6] still found Bmode-2D-US to be less accurate than 3D methods.

### 1.3. Novel Approach

In the comparison of different modalities for measuring thyroid volume, namely Bmode-2D-US, tracked 3D-US with different tracking methods, MRI and CT, the present study aims to (a) provide volume accuracy versus ground truth for all four modalities and all used 3D-US tracking technologies, and (b) report intraobserver and interobserver variability for Bmode-2D-US and 3D-US (all variants).

To address limitations in the state of the art, a multi-modality thyroid phantom with multiple anatomical samples is created for this study. This allows accurate measurement of ground truth volumes and surfaces, maintains a stable volume over time, and avoids unnecessary imaging and radiation exposure of patients.

## 2. Materials and Methods

### 2.1. Thyroid Phantom Creation

#### 2.1.1. Molding of the Samples

**Figure 1:**
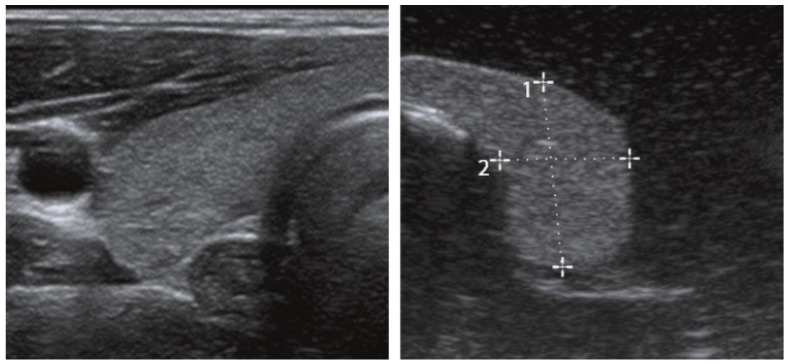
Combination image: (**left**)—human thyroid lobe as seen in ultrasound, (**right**)—thyroid phantom as seen in ultrasound with overlaid line measurements.

The shape of the thyroid can directly influence the quality of estimation. Most clearly, in the ellipsoid method, different thyroid shapes with quite different volumes could still produce the same line measurements, erroneously giving the same volume estimate. It is therefore critical to use a realistic thyroid shape in the phantom (Figure 1) for meaningful results.

**Figure 2:**
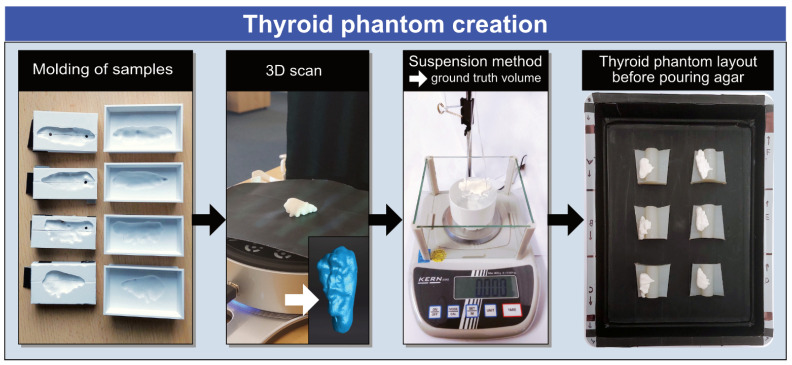
Creation of the anatomical thyroid phantom samples.

The thyroid lobe samples in the phantom were each separately molded from three-part 3D-printed polylactic acid molds (Figure 2) to preserve the thyroid shape. The shape of the molds was constructed using segmented MRI Data from the open-source SegThy dataset [7]. The six samples in the created phantom correspond to the left and right thyroid lobes of individuals 1, 2, and 14 in the SegThy MRI dataset.

#### 2.1.2. Suspension Method and 3D Scanning

In the present study, two different methods are applied to all sample volumes. One establishes the ground truth volume before insertion into the phantom; the other verifies it rigorously using a different methodology.

The suspension method described by Hughes et al. [8] makes use of digital scales to accurately measure displaced water volume. It is well established for the measurement of volume of anatomical structures [9]. This measurement technique was used as the ground truth for the volume of the samples (Figure 2). Calibrated digital scales (EMS 300-3, KERN & SOHN GmbH, Balingen, Germany) were used. This technique is not ideal for repeat measurements, as the surface is wetted each time.

Moreover, 3D scanning was used to verify the ground truth volume and was performed before the suspension measurement and insertion into the phantom. Additionally, this method generates an accurate mesh of the samples’ surface. Three-dimensional scanning is performed with an EinScan-SP (SHINING 3D, Hangzhou, China).

To further verify that the ground truth volume is indeed stable within the phantom over time, the suspension and 3D scanning measurements are repeated 31 days after phantom creation. Herein, the samples are carefully retrieved from the phantom.

#### 2.1.3. Phantom Assembly

The container in which the phantom is embedded should ideally cause few artifacts in the required imaging modalities while keeping the phantom stable from deformation and mechanical damage. A standardized euro container (600×400×75 mm, PP-C) was chosen to house the phantom. The inside was lined with rough black fabric tape to help reduce ultrasound imaging artifacts. To fix samples in the phantom, trachea-shaped pedestals were used as a footing for the samples to increase the familiarity of the ultrasound image and to provide orientation (Figure 2). The shaped pedestals were manufactured of the same agar mixture as the background material (Table 1) by molding in paper cups and subsequent cutting with a 3D-printed profile, creating a surface with a protruding 22 mm diameter half-circle. Fixing the samples with a small amount of cyanoacrylate adhesive was found to be a practical means of stabilization. As shown in Figure 1, the hardened adhesive on the trachea-shaped pedestals imitates the appearance of tracheal cartilage in ultrasound.

#### 2.1.4. Tissue-Mimicking Material

Phantoms are commonly made using tissue-mimicking material (TMM). The TMM composition is often chosen for a specific modality and imitated tissue type, while compatibility with other modalities is disregarded. Multi-modality phantoms are more complicated, as additives can simultaneously change the imaging properties in different imaging modalities.

Various synthetic materials can be used for multi-modality phantoms [10]. Despite its perishability, agar gel is also a common choice. To prepare a basic agar phantom, agar powder is mixed in heated water and cooled down to form a set agar gel. Specific additives can adjust TMM properties such as scattering, density and relaxation times for ultrasound, CT, and MRI respectively.

King et al. [11] constructed an agar phantom with a TMM composition that is standardized by the International Electrotechnical Commission for ultrasound phantoms [12] and applied it across US, MRI and CT. The additives are glycerol, silicon carbide, aluminium oxide and benzalkonium chloride.

While using ex-vivo anatomy within phantoms can be a good choice in certain circumstances [9], this approach was not chosen here. Initially, a preliminary phantom was created to investigate the option of using the approach of embedded ex-vivo anatomy, using thyroid-shaped turkey breast pieces. It was observed in ultrasound, CT, and MRI. Critically, the volume of the samples was observed to change considerably over time within the agar. Pouring warm agar onto the samples was also suspected to cause volume changes. This approach was therefore deemed unsuitable for this study.

A research group investigated the influence of different concentrations of agar and SiO_2_-powder for MRI and ultrasound, showing that agar concentration influences T1 and T2 relaxation times in MRI [13] as well as attenuation in ultrasound [14]. Using 6% *w*/*v* agar for the background and 6% *w*/*v* agar with 4% *w*/*v* SiO_2_ for a tumor model, ultrasound, CT and MRI showed good contrast and imaging properties close to soft tissues [15]. From the same research group, a molded thyroid phantom for MRI-guided ablation [16] likewise used 6% *w*/*v* agar for the background, 6% *w*/*v* agar with 4% *w*/*v* SiO_2_ for the thyroid and a hollow ABS tube as trachea. The simplicity of the research group’s approach, using a relatively small number of ingredients, represents a practical advantage.

Huber et al. [17] investigated an agar mixture in ultrasound, CT and MRI. To adjust appearance in ultrasound and MRI, 1.17% *w*/*v* agar was used for the background and 3.64% *w*/*v* agar for the sample. 0.8% *w*/*v* NaCl was used for realistic coil loading in MRI. The chosen composition of the TMM for the phantom in the present study is shown in Table 1. Agar was used both for the background and the thyroid. Based on initial experiments with different agar concentrations, using 3% *w*/*v* agar in the background and 6% *w*/*v* agar in thyroid samples proved suitable.

For the thyroid phantom in this study, TiO_2_ powder was used in the samples. It was chosen to adjust the optical properties of agar, normally translucent, to be matte-white opaque at a relatively low concentration, which enabled accurate 3D scanning of the sample surface. The appearance of agar in ultrasound not only depends on concentration but is severely affected by the temperature during preparation. Keeping the agar mixture at a temperature below 70 °C during preparation results in increased scattering [17].

By carefully adjusting the concentration of agar, NaCl and gadolinium contrast agent, phantom composition can be adjusted to closely mimic properties of specific tissues in MRI [18]. No recommendation was given for thyroid tissue. Gadoteridol was used in the phantom to enhance T1 contrast. The gadoteridol concentration in the current phantom was approximated from the T1 relaxation time of thyroid tissue [19] and its relation to concentration [20,21].

### 2.2. Data Acquisition

#### 2.2.1. Two-Dimensional B-Mode Ultrasound

In Bmode-2D-US, the ellipsoid method can be used to estimate volumes. In clinical volume estimation of the human thyroid lobes, it is the predominantly applied method. In its usage, the imaging plane of the ultrasound probe is aligned in the transversal plane to measure maximum width and depth of the thyroid lobe. For the third measurement, the probe is then rotated 90° to measure the maximum length of the lobe (cranial to caudal). The volume estimate is then given by $V=L_{max}\times W_{max}\times D_{max}\times f$, where the ideal factor *f* is reported in the literature to be 0.479 [22] or 0.529 [23]. The thyroid volume was calculated using an ellipsoid correction factor of f=0.5 throughout this study, consistent with common clinical practice. This method offers practical advantages in clinical application; however, its use in thyroid lobe volumetry reportedly leads to high inaccuracy [3,6,7].

In the present study, the ultrasound system Mindray M9 (Mindray, Shenzhen, China) is used with the linear ultrasound transducer L12-4S (3–13 MHz). The image settings (D 6.0; G 36; FR 47; DR 125; GrayMap 2; ExFOV 2) of the system were fixed for all observers and technologies in order to mimic the contrast and grey value of a human thyroid in the ultrasound image. With the used frame rate of 47 fps and an approximate normal free-hand scanning speed of 16 mm/s (estimate from prior clinician scan data) of the ultrasound probe, 2.9 frames are available for every 1 mm. The extended field of view setting (ExFOV) was chosen to increase the width of the image. It causes a curved lower border of the US image.

#### 2.2.2. Electromagnetic Tracking

**Figure 3:**
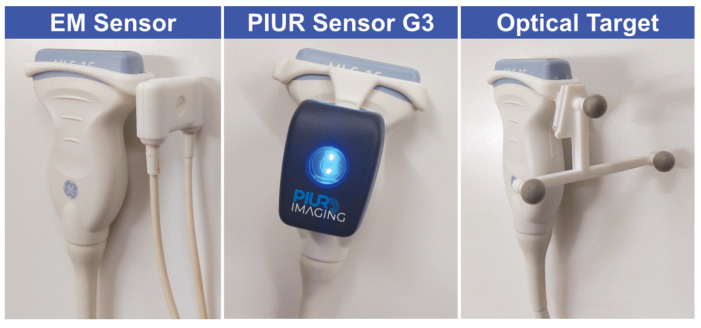
Add-ons for ultrasound probe in free-hand tracked ultrasound technologies.

**Figure 4:**
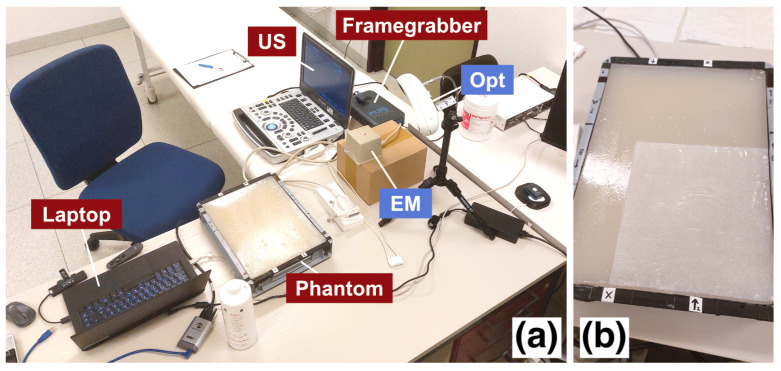
Experimental setup for tracked ultrasound methods. (**a**) EM field generator (EM) and camera for optical tracking (Opt); (**b**) ultrasound-gel-soaked fabric wipe as a structured phantom.

For electromagnetic tracking of the ultrasound probe, two six-degrees-of-freedom sensors are fixed relative to the ultrasound probe to track changes in the field (Figure 3) created by a field generator (Figure 4) aligned towards the working area. Each sensor has its own cable that leads into an interface unit. The 3D Guidance trakSTAR (Northern Digital, Waterloo, ON, Canada) is used for electromagnetic tracking.

#### 2.2.3. Inertial Tracking

Tracking without an external setup is an advantage of inertial tracking compared to electromagnetic and optical tracking. The PIUR G3 sensor (PIUR Imaging, Vienna, Austria) is one example of an inertial tracking system. The technology uses a wireless sensor that is fixed to the ultrasound probe (Figure 3) and a frame-grabber that captures the US image over an HDMI port. Gyroscopic data is recorded. For the PIUR G3 sensor, tracking is a combination of gyroscopic data and x-y movement from optical flow data. The optical flow sensor measures the relative movement of the ultrasound probe over the patient’s skin.

#### 2.2.4. Optical Tracking

In ultrasound with optical tracking, a camera setup is used to track movements of the ultrasound probe by tracking an attached optical target (Figure 3). Advantageously, no cable connection is required for the add-on. The line of sight between the camera and the optical target fixed to the ultrasound probe must be maintained. In the present study, the Polaris Vicra Position Sensor (Northern Digital, Waterloo, ON, Canada) is used for optical tracking.

#### 2.2.5. Computed Tomography

The phantom was scanned with CT using the neck protocol on NAEOTOM Alpha (Siemens Healthineers, Forchheim, Germany) with 1.5 mm slice thickness and a soft-tissue convolution kernel (Br40).

#### 2.2.6. Magnetic Resonance Imaging

MRI imaging of the phantom was performed with MAGNETOM Aera (Siemens Healthineers, Erlangen, Germany) at 1.5 T with 1.2 mm slice thickness. The chosen MRI sequence was a volumetric interpolated breath-hold examination sequence.

#### 2.2.7. Acquisition Procedure

The measurement setup for the four ultrasound-based methods is shown in Figure 4. Six observers acquired scans for each of the six samples using Bmode-2D-US and all three tracked 3D-US technologies. None of the observers had previous experience with the tracked ultrasound methods. Each received a short 1–2 min training on the usage of the different technologies. Four of the observers were radiologists, and two observers were nuclear medicine physicians. The nuclear medicine physicians had experience with thyroid ultrasounds (thyroid ultrasound experts: TE1 and TE2), while the radiologists had general ultrasound experience (ultrasound users: UU1, UU2, UU3 and UU4). Two observers (TE2, UU2) repeated the entire measurement set a second time. For the ultrasound-based methods, the ordering of technologies was randomized.

For CT and MRI, only one scan was acquired for the entire phantom. CT was performed the first day after the phantom creation. The data for the ultrasound-based methods was acquired on the second and third day. MRI was performed on day 11 due to logistic issues, while the repeated ground truth measurement was performed 31 days after the phantom creation.

### 2.3. Segmentation

**Figure 5:**
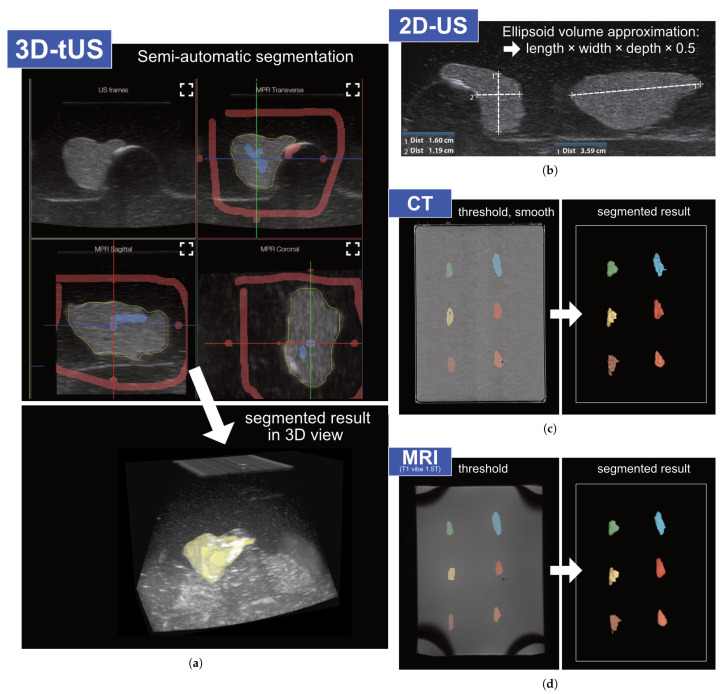
Volume measurement and segmentation process. White arrows correspond to data output. (**a**) Tracked 3D-US: semi-automatic segmentation of volume with binary colouring of tomographic ultrasound data within the PIUR tUS software; (**b**) Bmode-2D-US: measurement for volume estimation of a sample in Bmode-2D-US; (**c**) CT: segmentation of all samples from CT scan; (**d**) MRI: segmentation of all samples from MRI scan.

The acquired imaging data must be segmented to determine volume. The software application PIUR tUS 3.2.1 (PIUR Imaging, Vienna, Austria) was used for semi-automatic segmentation of the scans from the EM-3D-US and IMU-3D-US technologies (Figure 5a). Since PIUR tUS software forms an integral part of the PIUR acquisition and reconstruction workflow, it cannot readily be applied to imaging data acquired using other modalities. Accordingly, the software application 3D Slicer (version 5.03) [24] was used to manually segment Opt-3D-US, CT and MRI acquisitions assisted by initial thresholding. For CT and MRI, thresholds were selected manually rather than by any fixed criterion, as absolute threshold values are phantom-specific and do not generalize well. For Opt-3D-US, segmentation was semi-autonomous, using a manual binary coloring hint as input, which was edited sequentially to improve the segmentation output following the same approach used within PIUR software (PIUR tUS, version 3.2.1). Smoothing (2 mm median kernel) was applied to CT segmentations to address high-frequency noise along the borders of the thyroid phantom and to fill small holes. MRI segmentations were not smoothed, as the modality already exhibited inherent blurring at the borders. The observers were blinded towards the ground truth and towards the measurements of other observers. Although the segmentation was semi-automatic, all segmentations required observer interaction, manual editing where necessary, and final approval by the observer.

### 2.4. Statistical Methods

For statistical analysis, modified Bland–Altman plots [25] are used, where the *x*-axis is the ground truth instead of the mean, as is recommended when available [26]. This rests on the assumption that the ground truth estimates the true sample volume far more accurately than an average that incorporates less precise data, which could obscure interpretation. The differences must be reported both in absolute (${\mathrm{cm}}^{3}$) volume differences and in standardized (%) volume differences because it is possible that for some of the measurement methods the measurement error correlates with the true volume value [27]. Additionally, the 95% limits of agreement of ${\mathrm{Diff}}_{GT}$ for a number of *a* observers with *b* measured samples is calculated using the estimate for standard deviation (SD) [28].

The following formulas are used for calculation:

- Difference to ground truth:
  (1) $${\mathrm{Diff}}_{GT}^{{\mathrm{cm}}^{3}}=V_{measured}-V_{GT}$$
  (2) $${\mathrm{Diff}}_{GT}^{\%}=\frac{V_{measured}-V_{GT}}{V_{GT}}\times 100\%$$
- Intraobserver variability:
  (3) $${\mathrm{Diff}}_{Intra}^{{\mathrm{cm}}^{3}}=V_{2nd}-V_{1st}$$
  (4) $${\mathrm{Diff}}_{Intra}^{\%}=\frac{V_{2nd}-V_{1st}}{V_{GT}}\times 100\%$$
- Interobserver variability:
  (5) $${\mathrm{Diff}}_{Inter}^{{\mathrm{cm}}^{3}}=V_{TE2}-V_{TE1}$$
  (6) $${\mathrm{Diff}}_{Inter}^{\%}=\frac{V_{TE2}-V_{TE1}}{V_{GT}}\times 100\%$$
- 95% limits of agreement of ${\mathrm{Diff}}_{GT}$:
  (7) $$\pm 1.96SD=\pm 1.96\sqrt{\frac{\sum_{i=1}^{a}\sum_{j=1}^{b}{\left(V_{ij}-GT_{j}\right)}^{2}}{ab}}$$

For the 95% limits of agreement, the ground truth volume replaces its approximation from the sample-specific average. Pooling across observer groups (TE, UU) was performed intentionally to estimate the overall variability expected across a clinically realistic range of observer experience, rather than within a single observer subgroup. Pooling across samples assumes comparable residual variability across phantoms. As discussed in Section 4.4, the observed sample-specific bias indicates that this assumption is only approximately satisfied and therefore represents a limitation of the pooled estimate. Within this framework, the reported estimate assumes that the observers included are representative of the range of experience levels encountered in clinical practice, and that repeated sampling of comparable observer panels would yield comparable limits of agreement; this generalization applies equally to the reported interobserver variability. For all observers, only their first measurement set was included in this estimate, so the number of measurements is the same for all observers to avoid bias towards one observer. To also present a statistic with weaker assumptions, the range between minimum and maximum measured values of the expert measurements is presented together with the limits of agreement.

As a supplementary, exploratory analysis, non-parametric paired comparisons were performed to complement the descriptive Bland–Altman estimates. For each modality, the first-measurement-session volumes were averaged across all six observers per sample, and the resulting per-sample ${\mathrm{Diff}}_{GT}$ values (*n* = 6) were used for pairwise Wilcoxon signed-rank tests between modalities, as well as for one-sample Wilcoxon signed-rank tests against a hypothesized median difference of zero (i.e., perfect agreement with GT) for each modality individually. Additionally, session 1 vs. session 2 measurements of the two repeat observers (TE2, UU2) were compared using paired Wilcoxon signed-rank tests, computed separately per observer (*n* = 6). Given the small number of paired samples (*n* = 6 per test), the smallest attainable two-sided *p*-value is 0.03125, corresponding to all six paired differences pointing in the same direction. These tests are therefore reported as a low-power, confirmatory complement to the descriptive analysis rather than as an independent source of high-confidence evidence, and no correction for multiple comparisons was applied given their exploratory character.

## 3. Results

The ground truth volume for the six samples (A–F) was evaluated using the suspension method, giving the results in Table 2.

**Figure 6:**
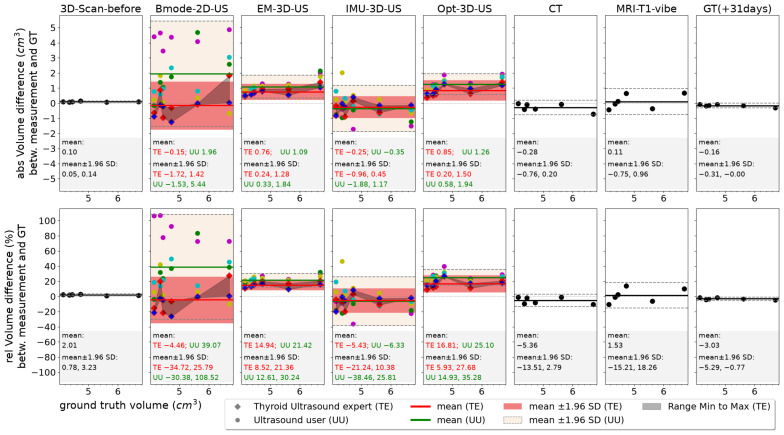
Difference from ground truth (GT): difference in measured volume by six observers to GT volume on *y*-axis and corresponding ground truth volume on *x*-axis. First scan used for value when repeated measurements were taken. Estimated limits of agreement (±1.96 SD) with the GT are shown as dashed line (no observer group) or colored (see legend). Mean is shown as solid black line or colored (see legend). Each color of the data points represents an observer. The ideal value of zero is marked with a dotted line.

**Figure A1:**
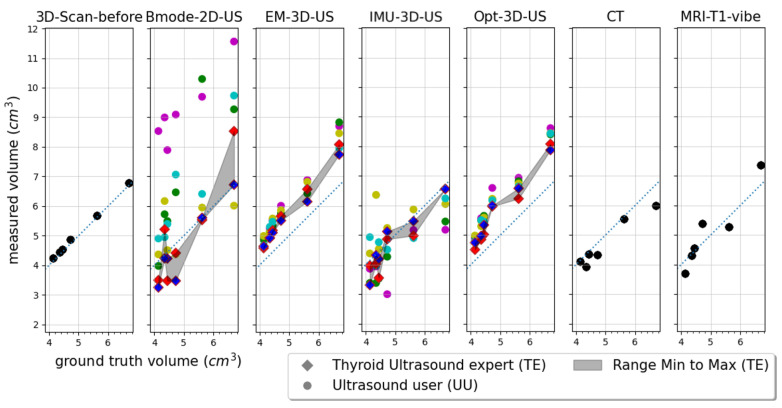
Measurements on the *y*-axis plotted against the respective ground truth volume on the *x*-axis. The first scan is shown when repeated measurements were taken. Marker colour denotes observer. Different markers are used depending on their experience level. The dotted line *y* = *x* shows the theoretical ideal.

**Figure A2:**
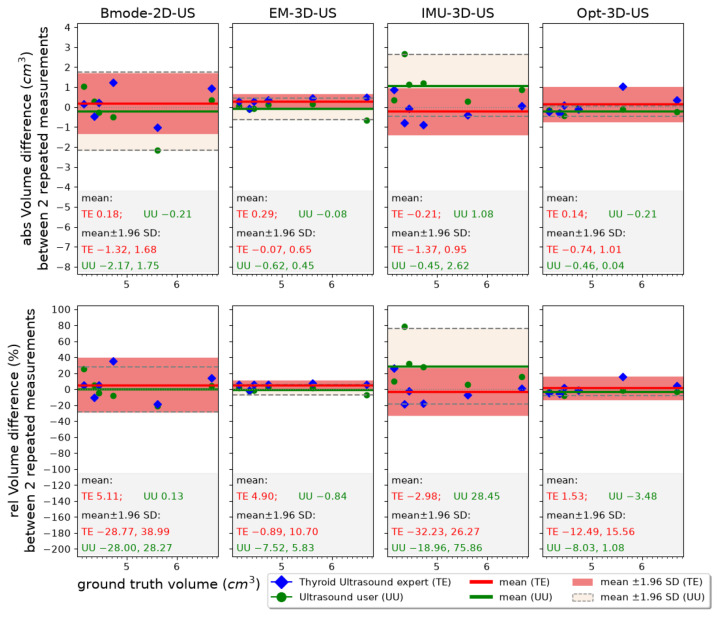
Intraobserver variability: difference in measured volume of observers from their own respective repeat measurement on *y*-axis and corresponding ground truth volume on *x*-axis. Estimated limits of agreement (±1.96 SD) with the respective repeat measurement are shown for different observer groups (see legend). The ideal value of zero is marked with a dotted line.

**Figure A3:**
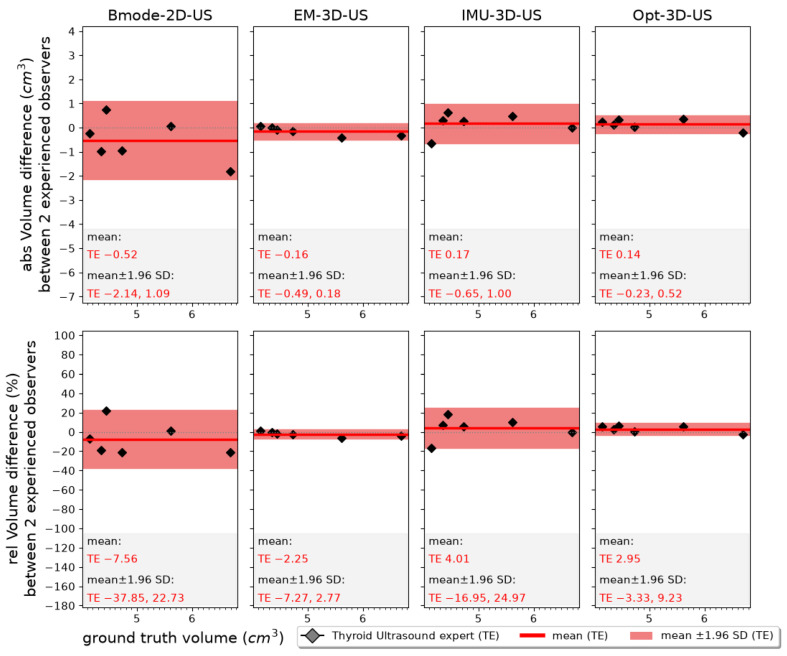
Interobserver variability: difference in measured volume between the two thyroid ultrasound experts on *y*-axis and corresponding ground truth volume on *x*-axis. Estimated limits of agreement (±1.96 SD) between the two experts are shown in red. The ideal value of zero is marked with a dotted line.

Figure 6, Figure A1, Figure A2 and Figure A3 show the different phantoms used in this study as points across the *x*-axis, where ground truth volume is measured. Data point shape represents the group affiliation (UU or TE), and colour represents different observers in the given plot. For Figure 6, Figure A2 and Figure A3, means and 95% limits of agreement (±1.96 SD) are given for the ground truth volume and observer groups used in the plot.

### 3.1. Difference Plot

Figure 6 shows the absolute difference ${\mathrm{Diff}}_{GT}^{{\mathrm{cm}}^{3}}$ and standardized difference ${\mathrm{Diff}}_{GT}^{\%}$ between the observers’ first set of measurements and the respective ground truth values of the samples. Values for means and 95% limits of agreement (±1.96 SD) are given in the boxes inside the plots. Measurements acquired for all modalities, including the 3D scan, are used in the comparison. The results show a clear difference for the different modalities. Bmode-2D-US showed the highest variation across observers, with a trend towards overestimation for the UU group, and underestimation for the TE group. For both EM-3D-US and Opt-3D-US there was a slight overestimation for both groups. For Bmode-2D-US the absolute volume difference mean was closest to ground truth for the TE group (−0.15 ${\mathrm{cm}}^{3}$ with ±1.96 SD resulting in −1.72 ${\mathrm{cm}}^{3}$ and 1.42 ${\mathrm{cm}}^{3}$), but furthest for the UU group (1.96 ${\mathrm{cm}}^{3}$ with ±1.96 SD resulting in −1.53 ${\mathrm{cm}}^{3}$ and 5.44 ${\mathrm{cm}}^{3}$). IMU-3D-US was the modality where the UU group was closest to ground truth in the mean (−0.35 ${\mathrm{cm}}^{3}$ with ±1.96 SD resulting in −1.88 ${\mathrm{cm}}^{3}$ and 1.17 ${\mathrm{cm}}^{3}$). Both CT and MRI showed high agreement with the ground truth (mean of −0.28 ${\mathrm{cm}}^{3}$ and 0.11 ${\mathrm{cm}}^{3}$), with MRI displaying a slightly higher deviation. Ground truth measurement after 31 days shows strong agreement with the original measured volume, with a mean difference of −0.16 ${\mathrm{cm}}^{3}$ and little deviation.

### 3.2. Formal Statistical Comparisons

As a supplement, exploratory analysis, pairwise Wilcoxon signed-rank tests [29] on the per-sample ${\mathrm{Diff}}_{GT}$ (averaged across observers, *n* = 6, Table A1) showed that IMU-3D-US, CT, and MRI did not differ significantly from one another (IMU-3D-US vs. CT: *p* = 1.000; IMU-3D-US vs. MRI: *p* = 0.219; CT vs. MRI: *p* = 0.438), and each differed significantly from Bmode-2D-US, EM-3D-US, and Opt-3D-US in all nine such comparisons (*p* = 0.031, the minimum attainable value at *n* = 6). Within the latter group, Bmode-2D-US did not differ significantly from either EM-3D-US (*p* = 0.219) or Opt-3D-US (*p* = 0.563); however, EM-3D-US and Opt-3D-US differed significantly from each other (*p* = 0.031), indicating that this group of three modalities was not fully homogeneous with respect to their agreement with GT. One-sample Wilcoxon tests against a hypothesized zero difference from GT (Table 3) indicated a statistically detectable systematic bias for five of the six modalities (Bmode-2D-US: median ${\mathrm{Diff}}_{GT}$ = +1.31 cm^3^, *p* = 0.031; EM-3D-US: +0.94 cm^3^, *p* = 0.031; Opt-3D-US: +1.02 cm^3^, *p* = 0.031; IMU-3D-US: −0.31 cm^3^, *p* = 0.031; CT: −0.24 cm^3^, *p* = 0.031). MRI was the only modality without a statistically detectable systematic bias (+0.04 cm^3^, *p* = 0.688). For the intraobserver comparison (session 1 vs. session 2, per-sample ${\mathrm{Diff}}_{GT}$, Table A2), UU2 showed a statistically significant difference between sessions for IMU-3D-US and Opt-3D-US (both *p* = 0.031), while TE2 showed no significant difference for any modality (*p* = 0.063–0.844).

### 3.3. X-Y Plot

Figure A1 in Appendix A shows measurements from the segmentations of the modalities used here plotted against the ground truth volume. The *x*-axis represents the ground truth value of samples, while the *y*-axis shows the measured volumes using the respective measurement technologies. From the X-Y plot, one can observe how measured values relate to certain ranges of likely true values, but the difference plot (Figure 6) allows the best overview of the results over all the given plots.

### 3.4. Intraobserver Plot

Figure A2 in Appendix A shows the absolute difference ${\mathrm{Diff}}_{Intra}^{{\mathrm{cm}}^{3}}$ and standardized difference ${\mathrm{Diff}}_{Intra}^{\%}$ of measurements taken by observers (TE2, UU2) from their own respective repeat measurement. Two repeated measurements for each ultrasound-based modality were compared for each phantom. For the expert observer, the modality with the mean value closest to ground truth was found to be Opt-3D-US, and EM-3D-US displayed the smallest standard deviation over all the measurements. For the observer from the UU group, EM-3D-US was the modality with the closest mean to ground truth, and Opt-3D-US displayed the smallest standard deviation range. Repeat measurement differences were mostly within a range of ±30%, with some outliers extending to nearly +80% for the UU group. Overall, EM-3D-US and Opt-3D-US showed the closest match between repeated scans, while the other modalities varied depending on user group and the phantom used.

### 3.5. Interobserver Plot

Figure A3 in Appendix A shows the absolute difference ${\mathrm{Diff}}_{Inter}^{{\mathrm{cm}}^{3}}$ and standardized difference ${\mathrm{Diff}}_{Inter}^{\%}$ of measurements between the first set of measurements of the two expert observers (TE1, TE2). Opt-3D-US showed the closest measurements for the mean value, with EM-3D-US and IMU-3D-US being very close. Bmode-2D-US showed clear differences between user measurements, leading to higher interobserver variability than the 3D-based variants. These results are based on the comparison between only the two expert observers (TE1 and TE2) and should therefore be interpreted as a limited estimate of interobserver variability.

## 4. Discussion

### 4.1. Quality of Ground Truth

The volume data from the 3D scan of the samples was shown to be consistent with the ground truth (suspension method), giving a difference close to zero (mean 0.10 ${\mathrm{cm}}^{3}$, SD 0.02 ${\mathrm{cm}}^{3}$). Therefore, the ground truth was accurate at the beginning. The CT measurement showed that the ground truth is likely also accurate during the time the samples were embedded within the phantom (mean −0.28 ${\mathrm{cm}}^{3}$, SD 0.24 ${\mathrm{cm}}^{3}$). The repeat measurement of the ground truth 31 days after the phantom creation showed that the ground truth was very consistent over time (mean −0.16 ${\mathrm{cm}}^{3}$, SD 0.08 ${\mathrm{cm}}^{3}$).

### 4.2. Influence of Experience Level in Ultrasound

Although the ellipsoid method for volume estimation in 2D ultrasound (Bmode-2D-US) is the most commonly used method in the clinical setting, the differences between measurements and the ground truth observed in this study were quite high, leading to a wide spread of results and inconsistent repeated measurements from the same observer (Figure A2). The difference in the accuracy between experience levels TE and UU (Figure 6) is the most pronounced in the ellipsoid method (Bmode-2D-US), as the UU placed measurements based on the longest diameters, as is done in the general case of the ellipsoid method, while the TE placed measurements in higher alignment with the body planes to estimate thyroid volumes and neglected the thyroid isthmus. Hereby, the UU had a consistent bias to severe overestimation of volumes in the ellipsoid method, while the TE had a small underestimation bias. Looking at the overall influence of experience level in the tracked ultrasound methods, the TE also consistently had smaller limits of agreement in ${\mathrm{Diff}}_{GT}^{{\mathrm{cm}}^{3}}$ than the UU. This most likely reflects the more controlled free-hand movements (steadiness and speed) of the TE. Consequently, the extent of the influence of experience level could be indicative of the robustness of the tracking method to free-hand movements. From the results, it is reasonable to assume that Opt-3D-US and EM-3D-US are the least sensitive to user movement quality. IMU-3D-US is more sensitive in comparison. Adequate operator training may therefore improve measurement consistency.

Intraobserver variability was lower for the 3D-tracked methods than for Bmode-2D-US. EM-3D-US and Opt-3D-US showed the tightest limits of agreement, while IMU-3D-US showed slightly higher variability but remained more consistent than the ellipsoid method. This suggests that 3D-tracked methods yield repeatable results once observers are familiar with the acquisition technique.

Due to their busy clinical schedule, both repeated observers were in more haste than for their first set of measurements and recorded faster scans. While the speed was measurably higher, their mean in the intraobserver plot seems little affected. It therefore seems likely that the methods have low dependence on scan speed. The order of the repeat measurement relative to the first was not randomized, and observers were not blinded to the fact that they were repeating a prior measurement session; both factors could introduce order effects that are not distinguishable from true intraobserver variability in the present design.

Bmode-2D-US showed the highest interobserver variability, reflecting the subjective nature of plane and endpoint selection in the ellipsoid method. The 3D-tracked methods demonstrated substantially lower interobserver variability, with Opt-3D-US showing the smallest mean difference and EM-3D-US and IMU-3D-US performing similarly. This indicates that segmentation-based 3D approaches provide more reproducible volume estimates across observers.

### 4.3. Comparison with the Literature

To contextualize the observer variability observed in the present study, Table 4 compares the standard deviation of ${\mathrm{Diff}}_{GT}^{\%}$ obtained here with values reported by Lyshchik et al. [3] and Freesmeyer et al. [6]. For Bmode-2D-US, the variability observed in the present study is closely comparable to that reported by Lyshchik et al., while both studies show substantially higher variability for Bmode-2D-US than for EM-3D-US, consistent with the general finding that tracked 3D-US reduces observer-dependent variability relative to the conventional ellipsoid method. Freesmeyer et al. reported somewhat lower variability across all modalities, including CT and MRI; the present study cannot determine the specific reason for this difference, as several factors, including differences in phantom design, observer training, segmentation protocols, ground truth measurement and study design, differ between the cited studies and the present work. Direct comparison across studies should therefore be interpreted cautiously.

### 4.4. Discussion of Sample-Specific Bias

Notably, a sample-specific bias appears in the difference plot (Figure 6) for EM-3D-US, IMU-3D-US, Opt-3D-US, and MRI. It can be most clearly seen when looking at the shape of the grey Min-to-Max range (Figure A1 for the measurements of the experts). Thyroid phantoms of different ground truth sizes are affected, ruling out a bias simply based on volume. Furthermore, it is unlikely to be an effect of incorrect ground truth since ground truth volume had not changed considerably, as confirmed by both the CT-based volume measurement shortly after phantom creation and the repeat suspension measurement after 31 days, neither of which followed the same sample-specific pattern as the observed bias. A possible reason for the bias might be that tracked ultrasound tracking errors are anisotropic and over-/underestimate differently on the specific shape and length in scan direction of the samples. A preliminary, non-quantitative comparison of relative length in scan direction, volume, and surface area appears to allow no trivial relation that captures the observed sample-specific bias, though this was not tested formally. Under this hypothesis, the similar bias observed for MRI would need to be attributed to coincidence, which could not be independently verified. Another possible source of sample-specific bias might be the effect of the specific shape on volume loss during segmentation smoothing operations. In a preliminary, non-quantitative comparison, applying different levels of smoothing to the 3D scan mesh of the samples did not appear to reproduce a pattern matching the observed sample-specific bias, though this was not tested formally.

### 4.5. Limitations Regarding Segmentation Software and Potential Bias

A methodologically important limitation concerns the fact that segmentation was not performed uniformly across modalities. Semi-automatic PIUR tUS software (PIUR Imaging, Vienna, Austria) was used to segment the EM-3D-US and IMU-3D-US acquisitions using an integrated, proprietary workflow, while CT and MRI were segmented through purely manual, threshold-assisted segmentation in 3D Slicer. Opt-3D-US followed the same conceptual approach as PIUR tUS (a manual binary coloring hint, edited sequentially), but this was replicated manually in 3D Slicer rather than performed within PIUR’s integrated software (Section 2.3). Consequently, the reduced inter- and intraobserver variability observed for EM-3D-US and IMU-3D-US should be interpreted as reflecting the performance of the complete acquisition-and-analysis pipeline rather than the tracking technology alone; part of this reduction may instead reflect the consistency and automation advantages of PIUR’s proprietary, integrated segmentation pipeline, compared to the same conceptual approach replicated manually for Opt-3D-US, which remained subject to the additional variability inherent in a non-integrated, manual reproduction of that workflow. The present study therefore cannot fully disentangle the respective contributions of the tracking hardware and the segmentation workflow to the observed reduction in observer variability. At the same time, segmentation software alone is unlikely to explain all observed differences between modalities. Image quality, spatial resolution, acquisition geometry, and tracking accuracy also influence the ease and reproducibility of segmentation. However, the present study design does not permit quantification of the relative contribution of these individual factors. A further consideration is the potential conflict of interest. Two authors (R.R., R.B.) are employees of PIUR Imaging, whose hardware (PIUR G3 sensor and IMU-3D-US) and software (PIUR tUS) were evaluated in this study. PIUR Imaging also provided the devices and technical support required for data acquisition. The fact that PIUR’s own integrated segmentation pipeline was applied to the modalities relying on PIUR’s own tracking hardware, while the same conceptual approach had to be manually reproduced for the Opt-3D-US modality, constitutes exactly the kind of asymmetry that a conflict of interest should raise concern about, independent of whether it demonstrably affected the results. To reduce the risk of bias, all image acquisitions and volume measurements were performed by clinicians not affiliated with PIUR Imaging, and the final analysis was reviewed by researchers independent of PIUR. Nevertheless, no independent, blinded re-analysis of the raw imaging data using a common segmentation implementation across all modalities was performed. Consequently, the influence of the tracking hardware cannot be completely separated from that of the segmentation implementation. Future studies should therefore apply a single, consistent segmentation strategy across all ultrasound-based modalities, for example, by using either fully manual segmentation or an identical semi-automatic workflow where technically feasible. Such an approach would allow the specific contribution of the tracking technology to observer variability to be evaluated more directly.

### 4.6. General Limitations

In the current evaluation of the results, a semi-automatic approach was chosen for the segmentation of recorded data. The more laborious manual planimetry approach might add accuracy but might be too time-consuming for some clinical scenarios. A fully manual segmentation approach might allow better investigation of the theoretical limits for the shown methods.

Due to the use of a phantom, different measurement methods experienced uneven difficulty based on how strongly they rely on the phantom’s realism, both at the tracking level and at the segmentation level. Among the tracking technologies, the tested inertial tracking system (IMU-3D-US) relies on the realism of the phantom’s surface, while EM-3D-US and Opt-3D-US do not. The IMU-3D-US system uses tracking from relative movements of the patient’s skin; here, the realism of the tracking result depends on the similarity of the phantom skin (Figure 4) to real patient skin, and potential differences in optical properties (texture, reflectance, etc.) between the phantom skin and actual human skin are a limitation of this study. The generalizability of the study result is furthermore limited by the small number of measurements and the limited number of observers. In particular, the reported interobserver variability is based on a direct comparison between only two expert observers (TE1, TE2), rather than the full six-observer panel, which substantially limits the precision and generalizability of this estimate. Furthermore, intraobserver variability was assessed using only one observer from each experience group (TE2, UU2), whose repeat measurements were not randomized in order or blinded against the first session, limiting the extent to which the observed variability can be generalized or fully attributed to measurement repeatability alone. The exploratory formal comparisons reported in Section 3.2 illustrate this limitation directly: with *n* = 6 samples, the smallest attainable two-sided *p*-value for any paired Wilcoxon test is 0.03125, so even the most consistent group-level differences observed here cannot be distinguished from a smaller or more nuanced effect with the present sample size. This reinforces that the study should be interpreted as a proof-of-concept phantom validation rather than as a definitively powered comparison between modalities. Since scan sessions for the ultrasound-based measurements were spread over only one day, any variability over time is not captured. Scan behavior of observers might, for example, be more similar when repeated on the same day than when repeated a longer time. The flat phantom geometry somewhat limits the realism that is achieved in the scanning trajectories, as they were limited to movements over a flat surface, while a more realistic 3D neck geometry might add further variability. The MRI acquisition was only possible 11 days after phantom creation due to an unexpected failure of our hospital’s scanner. Part of the error in the MRI volume may be caused by the resulting change in the phantom.

At the segmentation level, different imaging modalities experienced different reliance on phantom realism grounded in differing contrast between the thyroid sample and background material. This is expected to unevenly affect the realism of volume segmentation, potentially giving rise to biases linked to the surface area of samples; the extent of regions erroneously included in the thyroid volume due to similar contrast is likely also picked up to different extents across modalities.

### 4.7. Future Work

With the 3D scanning method of samples, the relation of measurement error with directionality or position on the sample could be closely investigated between methods. Herein, co-registration and the comparison with the 3D-scanned mesh could be performed for segmented data using the software CloudCompare (i.e., directional distance comparisons and distance comparison in direction of surface normals). Identifying the causes of error and their relative proportions will be key to improving these methods. A Pareto chart would likely be useful in the reporting of the magnitudes of individual causes of error.

After initial experiments, it was decided not to include thyroid nodules in the current version of the phantom to avoid obscuring the results of the main study. In an expanded study, molded thyroid nodules of several compositions could be embedded into the thyroid samples during creation, which would allow prior determination of ground truth for the nodules and clear nodule borders. To create nodules with unclear margins, a specific volume of unset agar could be injected during the thyroid molding process.

In future work, repeat segmentation with several software operators could show how the results depend on the segmentation process between operators. It is expected that some parts of the segmentation error might average out over different operators, while, to a certain extent, segmentation errors will be consistent throughout different segmentation software operators and show systematic biases related to the surface area of samples, as well as revealing other sample-dependent biases.

A similar or replication study would benefit from an increased number of observers with more repeat measurements over a longer period. This would increase statistical power for meaningful investigation of significant differences. The good stability of the phantoms’ ground truth over a 31-day timeframe observed in the present study enables future studies to prolong the measurement window beyond the more careful approach applied here. To overcome the limitations from the use of a phantom, a human subject study using all the presented ultrasound-based methods would be helpful.

## 5. Conclusions

This study compared thyroid lobe volumetry across six acquisition protocols using an anthropomorphic multi-modality phantom with anatomically realistic samples and a rigorously verified ground truth. Tracked 3D-US reduced both inter- and intraobserver variability relative to the conventional ellipsoid method in Bmode-2D-US, achieving variability comparable to CT and lower than MRI, while mean accuracy was similar across 3D-US, CT, and MRI. Bmode-2D-US remained strongly observer-dependent, with the influence of operator experience most pronounced for this method. Among the tracked methods, EM-3D-US and Opt-3D-US were the least sensitive to operator movement quality, whereas the inertial IMU-3D-US system showed somewhat higher sensitivity, indicating that adequate training is beneficial. These findings suggest that tracked 3D-US is a promising, radiation-free alternative for accurate and reproducible thyroid volumetry, warranting further evaluation in clinical practice, given that the limited reliability of the ellipsoid method currently constrains clinical decision-making. As these findings were obtained under controlled phantom conditions with a limited number of samples and observers, they should be regarded as preliminary proof-of-concept evidence rather than direct evidence for routine clinical implementation. Confirmation in a human subject study with a larger number of observers and repeated measurements over a longer period remains an important next step.

## Figures and Tables

**Table 1:** Components used for phantom creation.

Component	Sample	Background
Water	1000 g	1000 g
Agar	60.0 g	30.0 g
NaCl	9.0 g	9.0 g
TiO_2_	5.0 g	-
Gadoteridol 0.5 M	1.0 g	-

**Table 2:** Ground truth volume values for used samples.

A	B	C	D	E	F
4.14 ${\mathrm{cm}}^{3}$	4.44 ${\mathrm{cm}}^{3}$	5.62 ${\mathrm{cm}}^{3}$	4.35 ${\mathrm{cm}}^{3}$	4.73 ${\mathrm{cm}}^{3}$	6.70 ${\mathrm{cm}}^{3}$

**Table 3:** One-sample Wilcoxon signed-rank tests of ${\mathrm{Diff}}_{GT}$ against a hypothesized value of zero (i.e., perfect agreement with ground truth), based on per-sample means across all observers’ first measurement (*n* = 6 samples).

Modality	Median Diff_GT_ (cm^3^)	W	*p*-Value
Bmode-2D-US	+1.31	0.0	0.031 *
EM-3D-US	+0.94	0.0	0.031 *
Opt-3D-US	+1.02	0.0	0.031 *
IMU-3D-US	−0.31	0.0	0.031 *
CT	−0.24	0.0	0.031 *
MRI	+0.04	8.0	0.688

* *p* < 0.05. With *n* = 6 paired samples, *p* = 0.031 is the smallest attainable two-sided *p*-value.

**Table 4:** Standard deviation of ${\mathrm{Diff}}_{GT}^{\%}$ in %, comparison between present study and literature.

	Bmode-2D-US	EM-3D-US	CT	MRI
Present study (TE1, TE2)	15.4	3.3	4.2	8.5
Lyshchik et al. [3]	15.3	5.2	-	-
Freesmeyer et al. [6]	6.5–9.2	3.2–5.2	3.5–4.3	2.1–3.0

## Data Availability

The raw data require proprietary software for processing and are therefore available on request from the corresponding authors, who will provide access to both the data and the necessary tooling.

## Abbreviations

The following abbreviations are used in this manuscript:

abs	Absolute
Bmode-2D-US	B-mode two-dimensional ultrasound
CT	Computed tomography
EM	Electromagnetic
EM-3D-US	Electromagnetically tracked three-dimensional ultrasound
ExFOV	Extended field of view
GT	Ground truth
IMU-3D-US	Inertial-measurement-unit-tracked three-dimensional ultrasound
MRI	Magnetic resonance imaging
Opt-3D-US	Optically tracked three-dimensional ultrasound
rel	Relative
SD	Standard deviation
TE	Thyroid ultrasound expert
TMM	Tissue-mimicking material
3D-US	Three-dimensional ultrasound
US	Ultrasound
UU	Ultrasound user

## Appendix A. Additional Comparisons of Measurement Results

**Table A1:** Pairwise Wilcoxon signed-rank test *p*-values between modalities, based on per-sample ${\mathrm{Diff}}_{GT}$ averaged across observers’ first measurement (*n* = 6 samples).

	EM-3D-US	IMU-3D-US	Opt-3D-US	CT	MRI
Bmode-2D-US	0.219	0.031 *	0.563	0.031 *	0.031 *
EM-3D-US	–	0.031 *	0.031 *	0.031 *	0.031 *
IMU-3D-US		–	0.031 *	1.000	0.219
Opt-3D-US			–	0.031 *	0.031 *
CT				–	0.438

* *p* < 0.05. With *n* = 6 paired samples, *p* = 0.031 is the smallest attainable two-sided *p*-value.

**Table A2:** Intraobserver comparison: Wilcoxon signed-rank test *p*-values for ${\mathrm{Diff}}_{GT}$ between measurement session 1 and session 2, per repeat observer (*n* = 6 each).

Modality	UU2 (*n* = 6)	TE2 (*n* = 6)
Bmode-2D-US	1.000	0.688
EM-3D-US	1.000	0.063
IMU-3D-US	0.031 *	0.438
Opt-3D-US	0.031 *	0.844

* *p* < 0.05. With *n* = 6 paired samples, *p* = 0.031 is the smallest attainable two-sided *p*-value.
